# Microencapsulated phage composites with increased gastrointestinal stability for the oral treatment of *Salmonella* colonization in chicken

**DOI:** 10.3389/fvets.2022.1101872

**Published:** 2023-01-11

**Authors:** Bo Zhang, Yongxia Wang, Fangfang Wang, Yongying Zhang, He Hao, Xingbang Lv, Liuhang Hao, Yuxiang Shi

**Affiliations:** ^1^College of Life Science and Food Engineering, Hebei University of Engineering, Handan, Hebei, China; ^2^Engineering Research Center for Poultry Diseases of Hebei Province, Handan, Hebei, China

**Keywords:** *Salmonella*, microencapsulated phage, composites, stability, treatment

## Abstract

*Salmonella* infection, one of the common epidemics in the livestock and poultry breeding industry, causes great economic losses worldwide. At present, antibiotics are the most commonly used treatment for *Salmonella* infection, but the widespread use of antibiotics has increased drug resistance to *Salmonella*. Phage therapy has gradually become an alternative method to control *Salmonella* infection. However, phage, a specific virus that can infect bacteria, has poor stability and is prone to inactivation during treatment. Microencapsulated phage microspheres can effectively solve this problem. Accordingly, in this study, *Salmonella* phages were microencapsulated, using the xanthan gum/sodium alginate/CaCl_2_/chitooligosaccharides method, to improve their gastrointestinal stability. Furthermore, microencapsulated phages were evaluated for *in vitro* temperature and storage stability and *in vivo* therapeutic effect. Phage microspheres prepared with 1 g/100 mL xanthan gum, 2 g/100 mL sodium alginate, 2 g/100 mL CaCl_2_, and 0.6 g/100 mL chitooligosaccharides were regular in shape and stable in the temperature range of 10–30°C. Also, microencapsulated phages showed significantly improved stability in the simulated gastric juice environment than the free phages (*p* < 0.05). In the simulated intestinal fluid, microencapsulated phages were completely released after 4 h. Moreover, microencapsulated phages showed good storage stability at 4°C. In the *in vivo* experiments detecting *Salmonella* colonization in the intestinal tract of chicks, microencapsulated phages showed a better therapeutic effect than the free phages. In conclusion, microencapsulated phages exhibited significantly improved stability, gastric acid resistance, and thereby efficacy than the free phages. Microencapsulated phages can be potentially used as biological control agents against bacterial infections.

## Introduction

Salmonellosis causes serious economic losses to the livestock and poultry breeding industry. The diseases can be divided into two categories: one is typhoid and paratyphoid, and the other is acute gastroenteritis (1–3). *Salmonella*, a main food poisoning pathogen, may also cause related infectious diseases such as pullorum. In general, livestock and poultry are highly vulnerable to *Salmonella* infection (4), and the main sites of infection are the gut and organs (5). In case of compromised resistance, *Salmonella* enters the bloodstream through the intestines causing systemic infection increasing livestock mortality, and overuse/abuse of antibiotics (6). With the growing cases of drug resistance, the problem of drug resistance *Salmonella* has become a great concern. *Salmonella* epidemiological studies in the US and EU have shown that human and animal isolates do not have distinguished resistance at the molecular level, but once the antibiotic is approved for veterinary use, the multidrug-resistant *Salmonella* appears only a few years later (7–9). Lister and O'Brien et al. (10, 11) obtained through research that in the case of infection with *S*. enteritidis, high mortality was observed in chicks <2 weeks, sometimes as high as 20%, and some chicks in the affected chicken flocks showed signs of stunting. On the other hand, variations in the mortality rates were observed in experimental infections in chicks. Gast and Beard (12) suggested the presence of significant differences in the mortality rates (14.5–89.5%) in 1-day-old chicks orally inoculated with eight *S*. enteritidis isolates. Suzuki S et al. (13) found that *S*. enteritidis can cause chicks to be infected with salmonellosis and have a high mortality rate. Therefore, the search for a new method to prevent and treat *S*. enteritidis has attracted much attention.

Phage, a naturally widely existing bacterial virus that replicates and proliferates rapidly by infecting or lysing bacteria can be used for antibacterial effects (14–16). Phages with advantages such as strong specificity, safety and non-toxicity, self-replication, and widespread existence are expected to replace antibiotics against bacterial infections (17, 18). Phages have also been found effective against animal bacterial diseases but are prone to gastric acid and digestive enzyme degradation (19). Therefore, for sustained oral phage therapy, improving phage stability in the animal gut has become an important research issue.

Microencapsulation, a relatively new technology, has a very broad prospect (20). A drug/biological agent is microencapsulated by wrapping it with one or several substances (21) to achieve controlled release or stability (22). Though there are many microencapsulation studies, only a few are about the microencapsulation of *Salmonella* phage and therefore require more studies (23). Selecting a suitable coating material is extremely important to achieve the desired outcome of microencapsulation (24). Improper coating materials can reduce drug encapsulation or release rates (25). Moreover, microencapsulation materials must be non-toxic, acid resistant, and have good biocompatibility (26). Gelatin, whey protein, sodium alginate (SA), chitosan, chitosan oligosaccharides (COS), pectin, xanthan gum (XG), modified starch, etc. are the commonly used natural coating materials. Here, we prepared *Salmonella* SP4 microencapsulated phages using XG, SA, CaCl_2_, and COS to improve the antibacterial effect of phages.

## Materials and methods

### Bacteria, phages, and test animals

*Salmonella* Enteritidis (*S*. Enteritidis) CICC10467 [The strain CICC10467 is a common *S*. Enteritidis strain. Wang et al. (27) have proved through research that the amino acid sequence of FliC protein of strain CICC10467 and *S*. Gallinarum is identical, which will cause obvious *Salmonella* symptoms in chicks and increase the number of *Salmonella* colonization in chicks' intestines] was obtained from the China Industrial Microorganism Culture Collection Center, and phage SP4, isolated from sewage, was stored in the laboratory of Hebei Engineering University. Wastewater samples were collected from the wastewater treatment station of Hebei University of Engineering for phage separation. Approximately 100 mL of the sample was stored in sterile bottles and stored in a refrigerator (4°C) during transport to the laboratory for analysis. Centrifuge the wastewater at 6,000 rpm at 4°C for 15 min, and incubate it with the host bacteria at 37°C. A single plaque was chosen for purification for three passages with a specific host that showed a positive result, using a double-layer agar technique. Isolated plaque from the third purification passage was used to prepare 10-fold serial dilutions in SM buffer. Appropriate dilutions were used to prepare the overlay with the given host to yield the semi-confluent lysis and then harvest with 5 mL of SM buffer followed by centrifugation at 6,000 rpm for 15 min at 4°C. The supernatant was filtrated through 0.22-μm syringe filters, and phage lysates were kept at 4°C (28).

120 one-day-old Jingfen No. 1 laying hens (male and female 1:1) were purchased from Handan Xinguan Poultry Industry Co., Ltd (Handan, China). All animals were raised in an animal house with ad libitum feed and water. The study protocols (BER-YXY-2022027) were approved by the Institutional Animal Care and Use Committee and the Animal Research Ethics Committee of Hebei University of Engineering, China.

### Bacterial culture and phage propagation

*S*. Enteritidis CICC10467 was cultured in Luria-Bertani (LB) liquid medium at 37°C for 12 h (log-growth phase). For phage propagation, 20 μL of phage suspension was mixed with 500 μL of the *Salmonella* strain suspension in the logarithmic growth phase and 5 mL of LB medium containing 7 g/L agar; the mixture was incubated at 37°C overnight. The phage solution was filter sterilized using a 0.22 μm membrane filter.

### Preparation of microcapsules

Microencapsulation was performed as described by Ma et al. (29) with appropriate modifications for phages. Briefly, 1 g/100 mL XG solution was prepared by dissolving XG granules in deionized water. 2 g/100 mL SA solution was prepared in 50 mmol/L Tris-HCl buffer (pH7.5). The XG solution, SA solution, and propagated phage solution were mixed well in a beaker with stirring and vacuum degassing to remove air bubbles at 4°C. Next, the above-mentioned mixed solution was added dropwise using a sterile syringe to 2 g/100 mL CaCl_2_ solution (in deionized water) at 10 mL/min, and the reaction was allowed for 30 min at room temperature (RT). The phage microspheres were collected by filtration, washed with deionized water, and then moved to the 0.6 g/100 mL COS solution (in deionized water) for 30 min. After the COS treatment, the phage microspheres were collected by filtration, cleaned with deionized water, and some were stored in a closed container at 4°C. The other part of the microspheres was air dried in an oven at 30°C for 24 h and then stored in a closed container at 4°C. Phage titer was calculated as follows: Phage titer (PFU/mL) = plaque number × dilution factor × 100 (30). The phage encapsulation efficiency was calculated as follows: Encapsulation efficiency (%) = (amount of phage released from the dissolved microspheres/number of phages initially used to prepare the microspheres) × 100 (31). The results are expressed as the mean encapsulation efficiency ± standard deviation (SD) and each experiment had two replicates.

### Electron microscopy

Microspheres were examined for size and surface morphologies by scanning electron microscopy (SEM) at 5.0 kV (Hitachi S-4500; Hitachi Co. Ltd., Tokyo, Japan). The dried microspheres were mounted on metal grids using double-sided tape and coated with Au/Pd under vacuum. The diameter of phage microspheres was measured using an eyepiece micrometer and the calculation formula $\bar{d}$ = ∑d_i_/n (In the formula, $\bar{d}$ is the average particle size, ∑d_i_ is the sum of each particle size, and n is the number).

### Orthogonal design analysis of phage microencapsulation

Next, we performed an orthogonal test to explore the optimal concentration ratio of coating materials for the preparation of microencapsulated phages; the encapsulation efficiency was used as an index to evaluate the effects of XG, SA, CaCl_2_ and COS amounts on the encapsulation process. Orthogonal test factors were as follows: Factor A, XG concentration 0.5, 1, and 1.5 g/100 mL; Factor B, SA concentration 1, 2, and 3 g/100 mL; Factor C, CaCl_2_ concentration 1, 2 and 3 g/100 mL; Factor D, COS concentration 0.4, 0.6 and 0.8 g/100 mL (Table 1). All factor levels were selected based on previous literature (29). The experiment were repeated twice and the results are expressed as mean survival rate ± SD.

**Table 1:** Orthogonal test parameters of microencapsulated phage.

**NO**.	**A (%)**	**B (%)**	**C (%)**	**D (%)**	**Encapsulation rate (%)**
1	1	2	2	0.6	85.62 ± 2.14^d^
2	0.5	3	3	0.6	28.69 ± 1.69^a^
3	1.5	2	3	0.4	56.26 ± 2.64^bc^
4	1	3	1	0.4	29.61 ± 1.58^a^
5	1.5	1	2	0.6	35.68 ± 1.03^ab^
6	1	1	3	0.8	42.36 ± 1.95^b^
7	1.5	3	1	0.8	24.12 ± 2.36^a^
8	0.5	2	2	0.8	66.98 ± 1.74^c^
9	0.5	1	1	0.4	39.24 ± 1.41^b^
Factor primary and secondary: B > A > C > D					
Best plan: NO. 1					

(A) XG concentration. (B) SA concentration. (C) CaCl_2_ concentration. (D) COS concentration. The same lower letters represents no significant difference (P > 0.05) between the groups, different lower letters represent significant differences (P < 0.05) between the groups. Each value in the table represents the mean ± SD (n = 3).

### Temperature stability of phages

To determine the temperature stability, 1 mL of free phages (1 × 10^8^ PFU/mL) were placed in different microcentrifuge tubes, which were, respectively, incubated at 10, 20, 30, 40, 50, 60, and 70°C in a water bath. After 1 h of incubation, the samples were diluted 10-fold and used for phage titers determination using double-layer agar (29). For microencapsulated phages, 1 g of microencapsulated phages (1 × 10^8^ PFU/g) were placed in different microcentrifuge tubes, which were, respectively, incubated at 10, 20, 30, 40, 50, 60, and 70°C in a water bath. After 1 h of incubation, microsphere lysis solution (The microsphere lysis solution was composed of 14.7 g C_6_H_5_O_7_Na_3_·2H_2_O and 16.8 g NaHCO_3_ dissolved in 1 L SM buffer. Hebei University of Engineering, China) was added to each tube to crack open the microsphere, and the free particles were filtered through a 0.22 μm membrane filter. These free phage particles were then subjected to titer determination (29). The experiment were repeated twice and the results are expressed as mean survival rate ± SD.

### Gastric stability of phages

The gastric stability of free and microencapsulated phages was assessed using simulated gastric fluid (SGF) (32), which consisted of 3.2 mg/mL pepsin (Sigma-Aldrich, Oakville, Ontario, Canada) in 0.2% (wt/vol) NaCl, pH 2.0 and 3.0. Briefly, 1 mL of free phages (1 × 10^8^ PFU/mL) were added to a tube containing 10 mL of pre-warmed (37°C) SGF, and the tube was then incubated at 37°C for 10, 20, 30, 40, 50, and 60 min. 100 μL of the sample was collected at the respective time point and diluted 10 times for phage titer determination as described above. Because of prolonged survival time of microencapsulated phages in SGF, 1 g of phage microspheres (1 × 10^8^ PFU/g) were added to a tube containing 10 mL of pre-warmed (37°C) SGF and incubated at 37°C for 30, 60, 90, 120, 150, and 180 min. At the respective time, microencapsulated phages were lyzed and subject to titer determination (29). SM buffer mainly containing magnesium sulfate, sodium chloride and gelatin was used as a control, which is sterilized and used for diluting and preserving phage stock. The experiments were repeated twice and the results are expressed as mean survival rate ± SD.

### *In vitro* release assay of phages in simulated intestinal fluid

SIF was prepared as described in the US Pharmacopeia and consisted of 10 mg/mL pancreatin (Sigma-Aldrich, Oakville, Ontario, Canada) in 50 mM KH_2_PO_4_, pH 7.5 (33). Briefly, 1 mL of free phages (1 × 10^8^ PFU/mL) and 1 g of microencapsulated phages (1 × 10^8^ PFU/g) were added to a tube containing 10 mL of preheated (37°C) SIF, respectively, which were incubated at 37°C and 100 rpm. To investigate the release behavior of microencapsulated phages in SIF, 100 μL samples were collected at 0, 1, 2, 3, 4, 5, 6, 7, and 8 h, respectively. Like earlier, phage titer determination was conducted by the double-layer plate method (29). The experiments were repeated twice and the results are expressed as mean survival rate ± SD.

### Storage stability of phages at 4 and 25°C

To determine the storage stability, both kinds of phages were stored at 4 and 25°C, respectively. A small amount of free and microencapsulated phages was retrieved every 7 days and subjected to titer determination as described above (29). The experiments were repeated twice and the results are expressed as mean survival rate ± SD.

### Therapeutic evaluation of microencapsulated phages in infected chicks

The animals were randomly divided into three experimental groups (Groups B, C, and D) and one control group (Group A). This was repeated twice for each group of 10 chicks. All experimental groups were infected with *S*. Enteritidis CICC10467 (3 × 10^10^ CFU/mL, 0.5 mL/chicks) by oral gavaging. On the contrary, Group A (control) was fed with normal saline (0.5 mL/chicks). Group B was without any phage treatment. Group C and D were, respectively, treated with free (3 × 10^10^ PFU/mL, 0.5 mL/chicks) and microencapsulated phages (3 × 10^10^ PFU/g, 0.5 g/chicks) immediately after *S*. Enteritidis infection. Being in solid form, 0.5 g of microencapsulated phage was first weighed and then administered to chicks. All animals were raised in an animal house with *ad libitum* feed and water. After 7 days of the experiment, all animals were euthanized with carbon dioxide (Animal euthanasia equipment, SHANGHAI BIOWILL CO., Ltd.). The duodenum, jejunum, ileum, and colorectal tissues were collected and washed to remove intestinal contents. Each segment of the intestinal tube was weighed, crushed, and then continuously dilute the intestinal tube grinding solution and conduct *Salmonella* culture on XLD medium (XLD medium is a selective medium, which can be used to isolate *Salmonella*). It is well known that *Salmonella* presents black colony and *Shigella* presents colorless colony on XLD medium. In order to avoid false positive colonies on XLD medium. Therefore, black colonies grown on XLD medium are selected, the API 20E Enterobacteriaceae G-bacilli identification kit (for the identification of Enterobacteriaceae and other non fastidious gram-negative bacilli, Shanghai Xinzhong Biotechnology Co., Ltd., China) was used to conduct biochemical identification of bacteria according to the method of the user manual, and further verified to be *Salmonella*. The therapeutic effect of free and microencapsulated phages on infected chicks was estimated as the number of *Salmonella* colonization, which was calculated based on the number of *Salmonella* colonies on the XLD medium and the weight of the intestinal tube.

### Statistical analysis

The results were expressed as the means ± SD. All analyses were conducted using the GraphPad Prism 8 and SPSS Statistics 26 software. One-way ANOVA was employed to identify significant differences among multiple groups. ^ns^*p* > 0.05, ^*^p < 0.05, ^**^p < 0.01, ^#^*p* < 0.05, and ^##^*p* < 0.01. A difference was considered statistically significant at *p* < 0.05.

## Results

### Macro and microstructure of microencapsulated phages

**Figure 1:**
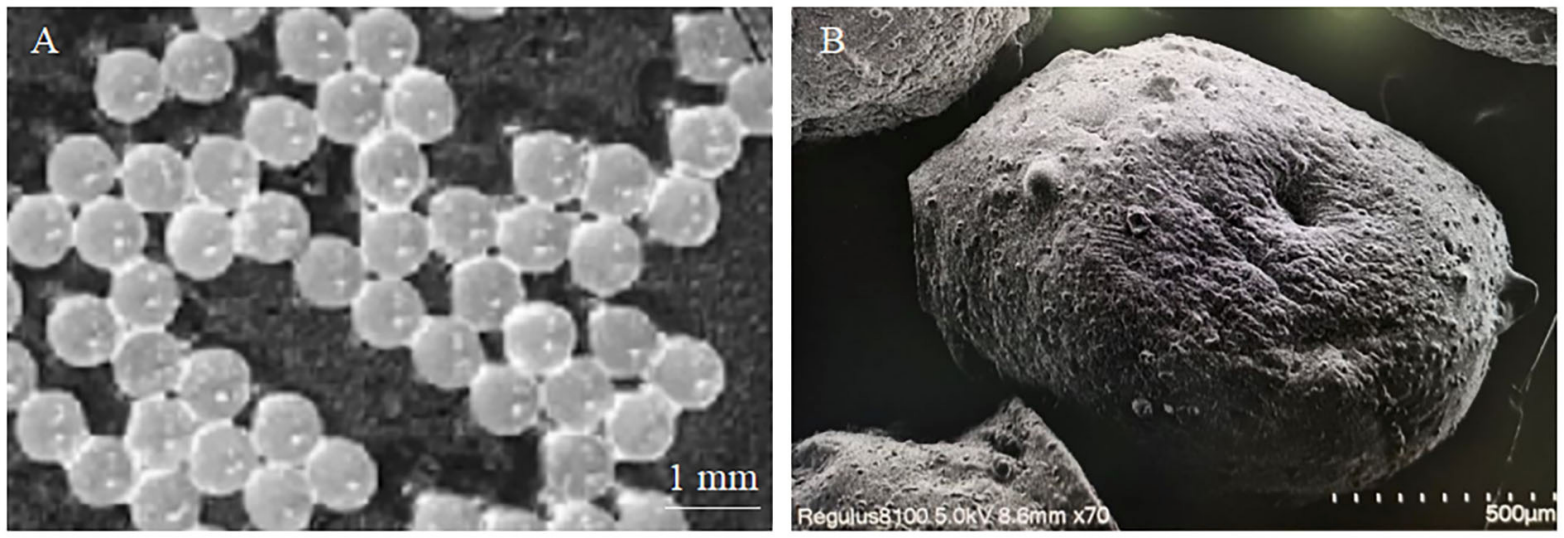
Observation image of microencapsulated phage. **(A)** Macroscopic image of microencapsulated phage. **(B)** Microscopic image of microencapsulated phage.

The microencapsulated phage microspheres were analyzed by a digital camera (SONY, ILCE-7RM3, 24.2 million pixels, China). The microspheres with circular outer surfaces had a uniform average size of 950 μm (range 930–970 μm) (Figure 1A). Because the microspheres need to be dried during SEM scanning, after drying, the microspheres will lose water and shrink, so the average size of the microspheres will become smaller. Microscopic morphological analysis of the microspheres was performed by scanning electron microscopy. The microspheres were generally spherical with a wrinkled surface and collapsed center; the average microsphere size was reduced to 350 μm (range 330–370 μm) (Figure 1B).

### Optimal microencapsulation parameters

The orthogonal analysis found that 1 g/100 mL XG, 2 g/100 mL SA, 2 g/100 mL CaCl_2_, and 0.6 g/100 mL COS produced the best encapsulation efficiency of 85.62 ± 2.14%. The least microencapsulation efficiency was 24.12 ± 2.36% (Table 1).

### Microencapsulation improved the thermal stability of phages

**Figure 2:**
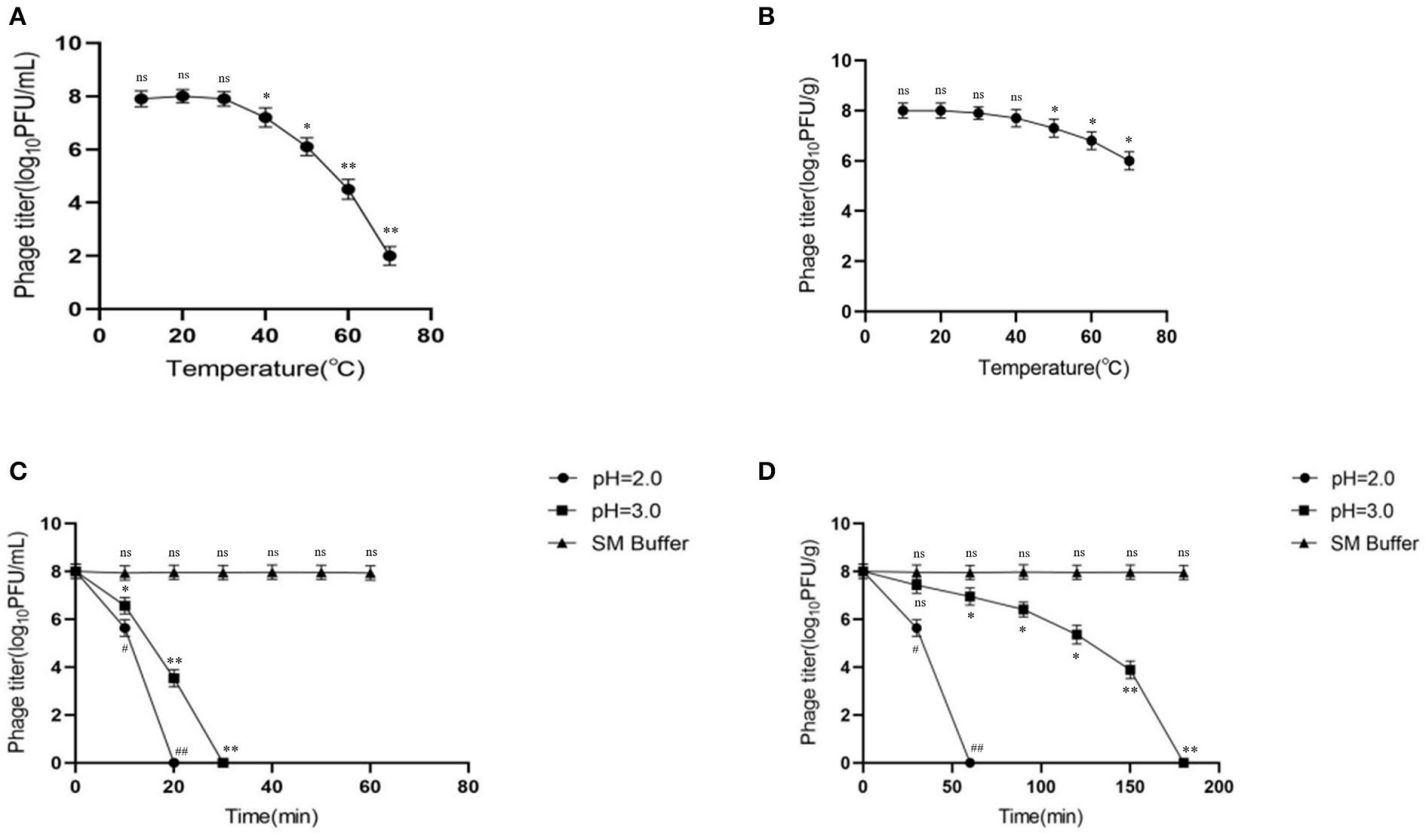
Stability of microencapsulated phage at different temperatures and SGF. **(A)** Effects of different temperatures on free phage. **(B)** Effects of different temperatures on microencapsulated phage. **(C)** The effect of SGF on free phage. **(D)** The effect of SGF on microencapsulated phage. ns represents significantly the same (*P* > 0.05); *represents significantly the different (*P* < 0.05); **represents significantly the different (*P* < 0.01); ^#^represents significantly the different (*P* < 0.05); ^##^represents significantly the different (*P* < 0.01). Each value in the figure represents the mean ± SD (*n* = 3).

Activity comparison revealed that microencapsulation improved the temperature stability of phages. At 10, 20, and 30°C, the activities of free and microencapsulated phages showed no difference, but a further increase in temperature (40–70°C) decreased their activities. However, compared with free phages, the activity of microencapsulated phages remained higher; at 70°C, the activity of free phages was 2 log_10_PFU/mL (Figure 2A), while that of microencapsulated phages was 6 log_10_PFU/g (*p* < 0.05) (Figure 2B). This data indicated that microencapsulated phages were more temperature resistant than the free phages.

### Microencapsulation improved gastric stability of phages

To determine the effect of microencapsulation on the gastric stability of phages, the microencapsulated phages were tested in SGF. At pH 2.0 and 3.0, the activity of free phages declined rapidly reaching inactivation at 20 and 30 min, respectively (Figure 2C). This indicated less gastric (acidic environment) stability of free phages. Interestingly, the activity of microencapsulated phages also decreased gradually but took longer to reach inactivation than the free phages (60 and 180 min at pH 2.0 and 3.0, respectively) (Figure 2D). Overall, these data indicated that microencapsulated phages were more stable in an acidic environment than the free phages. Also, there was no significant change in the activity of free and microencapsulated phages in the SM buffer.

### Microencapsulated phages showed good release in SIF

**Figure 3:**
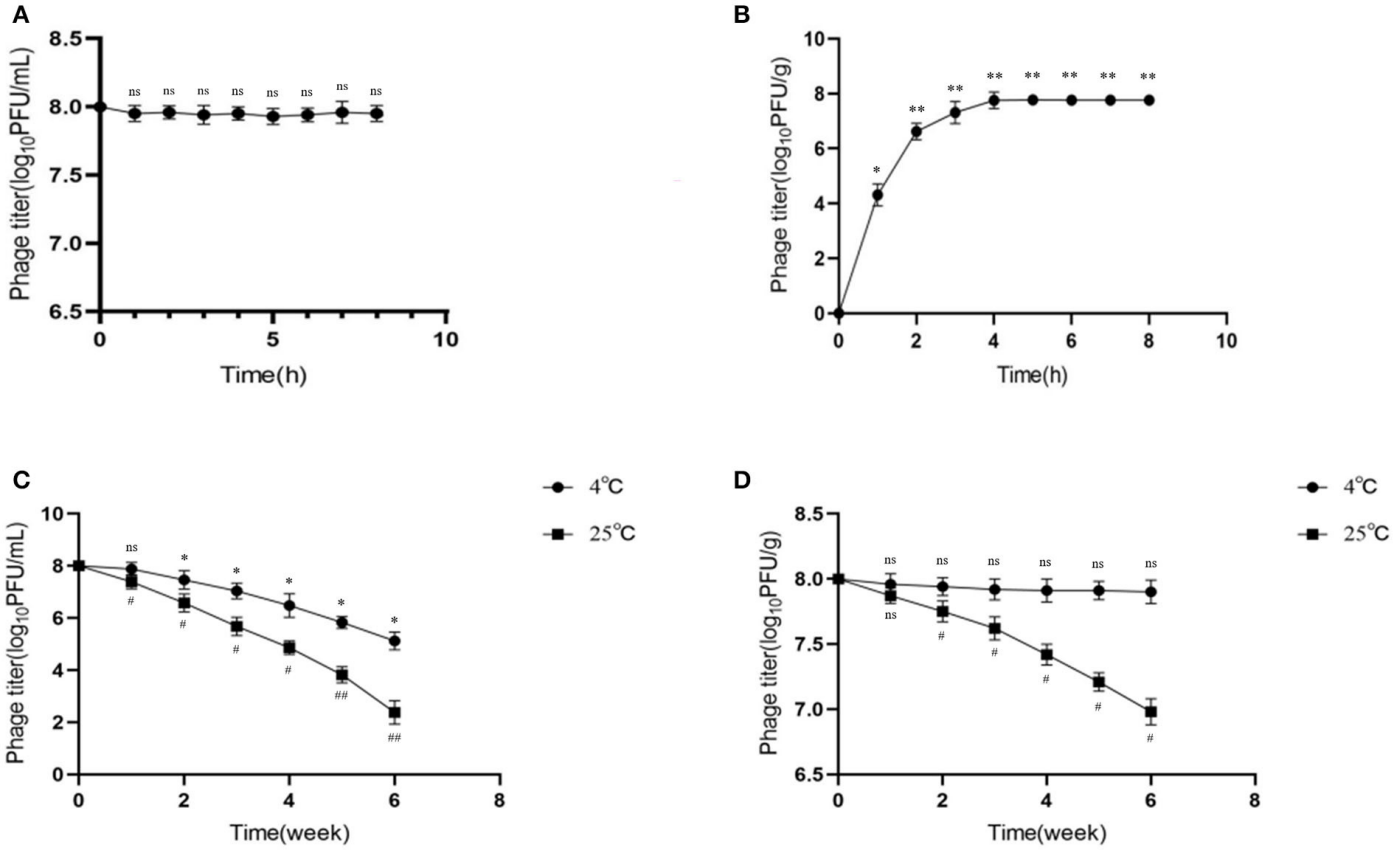
Release behavior and storage stability of microencapsulated phage in simulated intestinal fluid. **(A)** Stability of free phage in SIF. **(B)** Release behavior of microencapsulated phage in SIF. **(C)** Effects of 4°C and 25°C on free phage. **(D)** Effects of 4°C and 25°C on microencapsulated phage. ns represents significantly the same (*P* > 0.05); *represents significantly the different (*P* < 0.05); **represents significantly the different (*P* < 0.01); ^#^represents significantly the different (*P* < 0.05); ^##^represents significantly the different (*P* < 0.01). Each value in the figure represents the mean ± SD (*n* = 3).

Microencapsulated phage microspheres should not only improve gastric stability but also allow viable phage release at the desired site (e.g., the small intestine). Accordingly, we tested phage release in SIF by determining phage activity. The results showed that the activity of free phages (indicating the release rate) did not change significantly in SIF and was relatively stable over time (Figure 3A). When the microencapsulated phages were put into SIF, the microspheres began to swell and disintegrate, and the release rate stabilized after the burst effect. The phage titer gradually increased with time and after 4 h became stable (Figure 3B). This indicating that all the microencapsulated phages were released in SIF.

### Microencapsulation improved the storage stability of phages

Good storage temperature stability is necessary for long-term storage and application of microencapsulated phage microspheres. In this study, phages were examined for storage stability at refrigeration (4°C) and room incubation (25°C) temperatures. The results showed that the titer of free phage decreased at both 4 and 25°C, by 2.88 and 5.62 log_10_PFU/mL, respectively (*p* < 0.05) (Figure 3C); the decrease was more significant at 25°C. On the contrary, the activity of microencapsulated phages did not significantly change at 4°C (*p* > 0.05) but decreased by 1.02 log_10_PFU/g at 25 °C (*p* < 0.05) (Figure 3D). This data indicated better storage stability of microencapsulated phages over free phages; 4°C seems to be the good storage temperature.

### Pathological changes of organs and intestines in chicks infected with *Salmonella*

**Figure 4:**
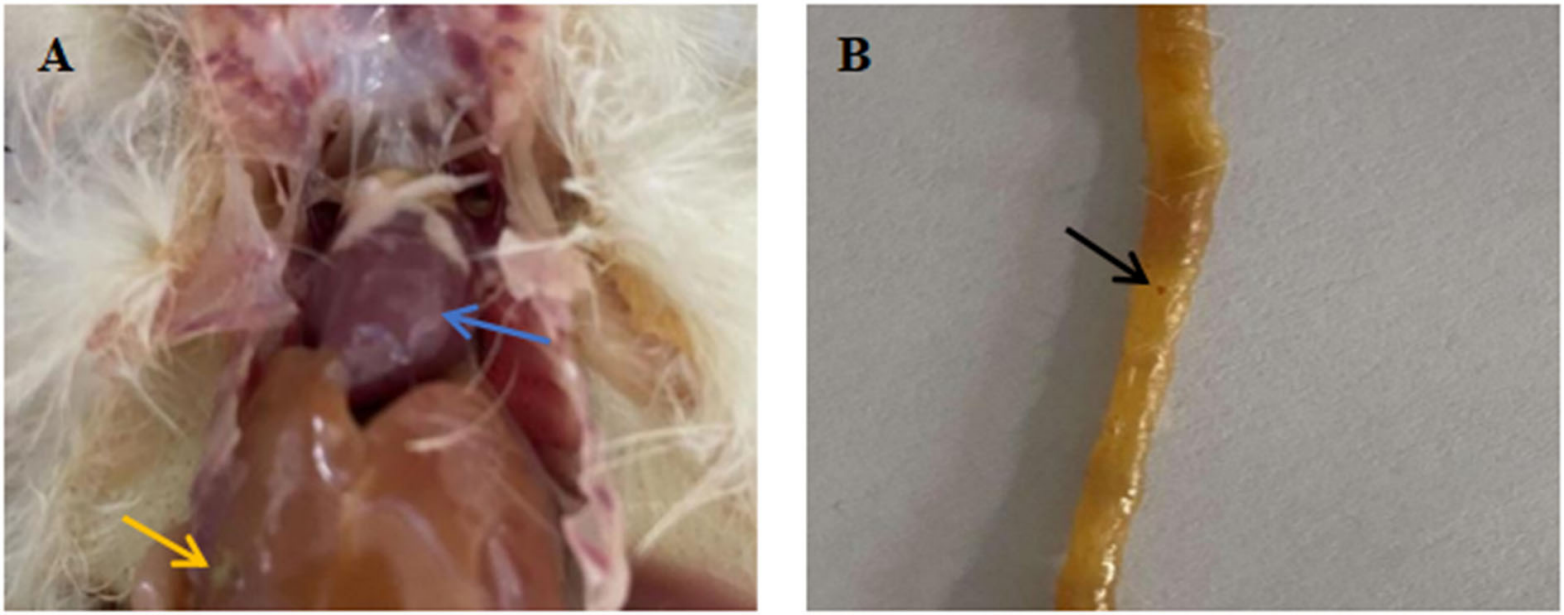
Pathological anatomy of organs and intestines of chicks infected with *Salmonella*. **(A)** Pathological anatomy of organs. **(B)** Pathological anatomy of intestine. Blue arrow: fibrous exudation on the heart surface. Yellow arrow: the liver is swollen, yellowish brown, with gray white necrotic foci. Black arrow: there are obvious bleeding spots on the intestinal tract.

Chicks infected with *Salmonella* showed obvious symptoms of salmonellosis. Necropsy showed fibrous exudation on the surface of the heart (Blue arrow), enlarged liver, which was earthy-yellow, and grayish white necrotic lesion on it (Yellow arrow) (Figure 4A). There was significant bleeding in the intestine (Black arrow) (Figure 4B).

### Microencapsulation improved the therapeutic effect of phages

**Figure 5:**
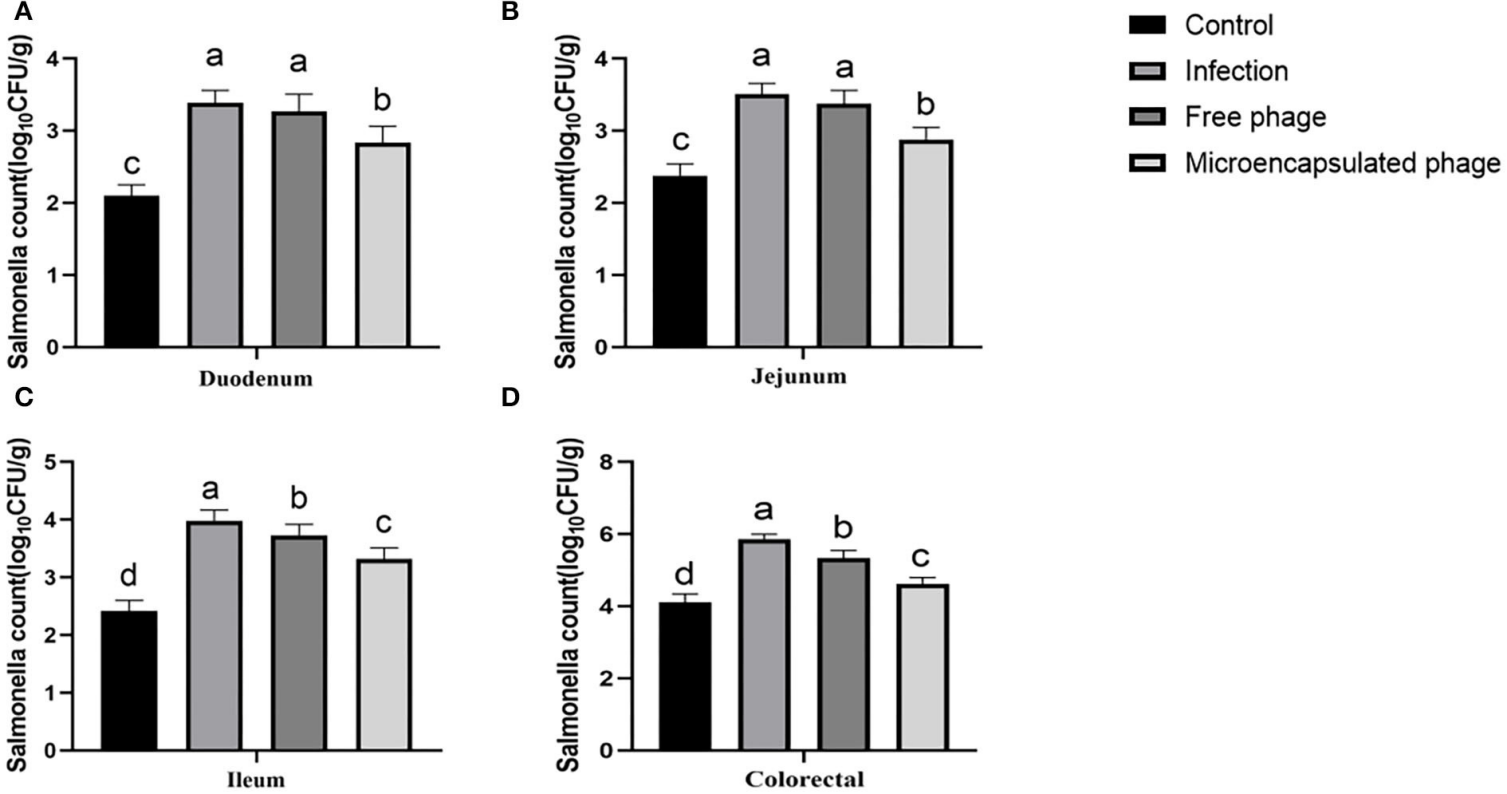
Therapeutic effect of microencapsulated phages on infected chicks. **(A)** Changes in the number of *Salmonella* colonization in duodenum. **(B)** Changes in the number of *Salmonella* colonization in jejunum. **(C)** Changes in the number of *Salmonella* colonization in ileum. **(D)** Changes in the number of *Salmonella* colonization in colorectal. The same lower letters represents no significant difference (*P* > 0.05) between the groups, different lower letters represent significant differences (*P* < 0.05) between the groups. Each value in the figure represents the mean ± SD (*n* = 3).

The microencapsulated phages showed improved temperature stability, gastric stability, good SIF release, and storage stability. Next, we tested their *in vivo* therapeutic effect against *Salmonella* in chicks. As described in the method section, the animals were divided into four groups: Group A (control: not infected), Group B (infected), Group C (infected and treated with free phages), and Group D (infected and treated with microencapsulated phages). Since *S*. Enteritidis mainly invades the intestine, the chick intestines (duodenum, jejunum, ileum, and colorectal tissues) from the respective groups were examined for the therapeutic effect of phages. Compared with the Group A, the number of *Salmonella* colonization from the duodenum, jejunum, ileum, and colorectal samples increased by 1.29, 1.13, 1.56, and 1.75 log_10_CFU/g in Group B (*p* < 0.05). Compared with the Group B decreased by 0.12, 0.13, 0.25, and 0.52 log_10_CFU/g after treatment with free phages in Group C (*p* < 0.05). Compared with the Group B decreased by 0.55, 0.63, 0.66 and 1.24 log_10_CFU/g after treatment with microencapsulated phages in Group D (*p* < 0.05). Compared with the Group C decreased by 0.43, 0.50, 0.41 and 0.72 log_10_CFU/g after treatment with microencapsulated phages in Group D (*p* < 0.05) (Figure 5). The therapeutic effect of Group D is better than that of Group C. These results indicated a better *in vivo* therapeutic effect of microencapsulated phages against the *Salmonella* infection.

## Discussion

Exposure to low pH, organic solvents, dehydration, and high temperature can irreversibly damage viruses (34). This study too showed that the viability of free phages was rapidly lost after exposure to SGF. Therefore, ensuring phage stability is a key consideration in designing efficient microencapsulation methods. Also, the microencapsulation process should employ mild physical conditions and use compatible materials that would not impair the biological activity of phages. Furthermore, when the goal is to deliver live phages to the gut, the encapsulating material should protect the phages from the acids and enzymes present in gastric juice and readily dissolve or swell in weakly alkaline intestinal media. The encapsulation process in this study was performed in a mild water-based environment and did not reduce the phage viability. Consistent with a previous report about encapsulation of probiotics (35), we also achieved high phage loading efficiency of 85.62%. When the CaCl_2_ droplets were mixed with phage-containing SA gel medium, the droplets instantly formed gel microspheres forming a three-dimensional network of ionically cross-linked alginate. In addition, our microencapsulated phages showed excellent stability when stored at 4°C. Spermine alginate and poly (D,L-lactide co glycolide) have been previously used for the microencapsulation of rotavirus, but only about 14 and 30% of the initial virus amount could be encapsulated, respectively (36). Therefore, it appears that the calcium alginate (CA) matrix is more suitable for phage encapsulation.

Studies have shown that directly coating phages with SA is not an ideal method, and such coating results in low efficiency of phage packaging (37). Here, we found that adding XG effectively improved this phenomenon. XG has good macromolecular special structure and colloidal properties and is often used as a microencapsulation material (38). Microspheres prepared from SA and CaCl_2_ are prone to tailing and irregular shapes (39). With the good colloidal properties of XG, SA combined with XG can significantly improve this problem and thereby encapsulation efficiency. SA, a natural polysaccharide, has good sensitivity, biocompatibility, and mild emulsification process. It is often used as a coating material and as a therapeutic agent in the veterinary field (40). The SA-CaCl_2_ system can efficiently realize the microencapsulation of phages; SA reacts with Ca^2+^ ions to form CA polymer, which has good biodegradability and biocompatibility. COS, the products of chitosan processing, have good compatibility, non-toxicity, and film-forming properties (41). COS are often used as a film-forming material and have been found better than chitosan. After COS reacts with CaCl_2_, microencapsulated phages improving their stability. These selected coating materials are inexpensive, simple, easy to obtain, and without harsh production processes, which is suitable for large-scale industrial production.

During microencapsulation, it is important to control the concentration of the coating material. A too low SA concentration results in a dilute mixture. On the contrary, a high SA concentration makes the mixture too viscous hindering the formation of microspheres. The system stability may decrease after SA solution mixing with phages. The combination of SA-XG helps form microspheres of regular shape and improves encapsulation efficiency.

The microencapsulation technique for biological preparations has been well established (42). Yin et al. showed that microencapsulation significantly improved the acid survival time of phages, which is consistent with our results (43). In this study, a variety of natural materials with different characteristics were used in combination, which overcame the deficiency of free phage the antibacterial therapeutic effect. Although microencapsulated phages also became inactive in SGF at pH 2.0 and 3.0, microencapsulation effectively prolonged their survival time compared with free phages. The thermal stability of free phages is poor, and they are vulnerable to high temperature environment, which is not conducive to the treatment and preservation of free phages (44). We showed that microencapsulated phage microspheres had better thermal stability than the free phages. Notably, the phage titer of the microencapsulated phage microspheres in deionized water remained unchanged indicating no release in deionized water. CA gel has pH-responsive properties. In the acidic gastric environment, the carboxyl groups on the sugar chains of CA get protonated, and hydrogen bonding occurs between the sugar chains. This forms a water-insoluble alginate gel on the surface of the microspheres, named the alginate shell. At the acidic pH, the CA gel structure does not swell and protects the embedded objects from gastric acid and digestive enzymes; however, in a neutral pH intestinal environment, the CA gel swells and releases the embedded objects (45). When the microencapsulated phages were tested for release in SIF, they were not completely released in a short time. This may be because in the beginning only a small amount of phage particles detached from the microsphere surface. However, in the later stage, the microsphere coating material fully reacted with intestinal fluid leading to a burst effect, and all phages were rapidly released. For clinical antibacterial applications, phages must survive the gastric juice and then release into the intestinal fluid. We showed that the chosen chemical coating material achieved the same and produced clinically effective microencapsulated phage microspheres.

Furthermore, the *in vivo* therapeutic effect of microencapsulated phages was tested against salmonellosis in chicks. The *Salmonella*-infected chicks were treated with free and microencapsulated phages and then *Salmonella* colonization was estimated from the intestinal tract tissues. Both free phages and microencapsulated phages can reduce the number of Salmonella colonization in chicken intestine. Compared with free phages, microencapsulated phages significantly reduced the *Salmonella* infection in chicks, indicating their higher anti-bacterial therapeutic efficiency. Ma et al. microencapsulated phages by using sodium alginate, CaCl_2_ and chitosan. The experiment proved that microencapsulated phages can effectively treat salmonellosis, which is consistent with the results of this experiment. The encapsulation efficiency of phages in this experiment is significantly higher than the above experiment (29). Yin et al. verified that free phages and microencapsulated phages could effectively reduce bacterial concentration through *in vivo* experiments in mice, and the effect of microencapsulated phages was better than that of free phages (43).

In conclusion, compared with free phage, XG-SA-CaCl_2_-COS microencapsulated phage microspheres exhibited significantly improved survival rates under simulated gastrointestinal conditions, and thermal and storage conditions, and microencapsulated phage can release phages in simulated intestinal fluid. More importantly, their therapeutic effect against salmonellosis also improved. The current encapsulation method can improve the effectiveness of phages for oral therapeutic applications. Future work may be necessary to explore other surface coating materials. The current phage microspheres allow long-term storage of encapsulated phages and their direct delivery to the intestine protecting from gastric conditions. Innovative microencapsulated phage therapy can be an effective alternative to antibiotics.

## Data availability statement

The original contributions presented in the study are included in the article/Supplementary material, further inquiries can be directed to the corresponding author.

## Ethics statement

The animal study was reviewed and approved by the Institutional Animal Care and Use Committee and the Animal Research Ethics Committee of Hebei Engineering University of China, the Research Scheme is BER-YXY-2022027.

## Author contributions

Conceptualization and writing–review and editing: YS. Data curation: BZ and YW. Formal analysis: HH and XL. Investigation: FW and YZ. Supervision: BZ and LH. Writing—original draft: BZ and YS. All authors contributed to the article and approved the submitted version.
