# Supplementary material for: Microencapsulated phage composites with increased gastrointestinal stability for the oral treatment of Salmonella colonization in chicken
Source: Front Vet Sci. 2023 Jan 11;9:1101872. doi: 10.3389/fvets.2022.1101872 (PMC9875011; doi:10.3389/fvets.2022.1101872)
Supplement: Supplementary file 1 [file Data_Sheet_1.PDF]

Table S1. Activity of free phages and microencapsulated phages at different temperatures

Group	10℃	20℃	30℃	40℃	50℃	60℃	70℃
Free phage	7.93±0.31	7.98±0.34	7.91±0.33	7.25±0.42	6.18±0.35	4.52±0.41	2.03±0.42
Microencapsulated phage	7.97±0.33	8.00±0.34	7.94±0.37	7.72±0.41	7.36±0.43	6.87±0.42	6.04±0.41

Table S2. Activity of free phages in simulated gastric juice

pH	0 min	10 min	20 min	30 min	40 min	50 min	60 min
2.0	8.00±0.30	5.64±0.35	0	0	0	0	0
3.0	8.00±0.30	6.57±0.34	3.54±0.36	0	0	0	0
SM Buffer	8.00±0.30	8.00±0.31	8.00±0.33	8.00±0.32	8.00±0.31	8.00±0.30	8.00±0.31

Table S3. Activity of microencapsulated phages in simulated gastric juice

pH	0 min	30 min	60 min	90 min	120 min	150 min	180 min
2.0	8.00±0.30	5.70±0.37	0	0	0	0	0
3.0	8.00±0.30	7.43±0.34	6.95±0.36	6.41±0.31	5.36±0.39	3.89±0.37	0
SM Buffer	8.00±0.30	8.00±0.30	8.00±0.32	8.00±0.31	8.00±0.32	8.00±0.31	8.00±0.30

Table S4. Activity of free phages and microencapsulated phages in simulated intestinal fluid

Group	0 h	1 h	2 h	3 h	4 h	5 h	6 h	7 h	8 h
Free phage	8.00±0.30	7.95±0.06	7.96±0.05	7.94±0.07	7.95±0.05	7.93±0.06	7.94±0.05	7.96±0.08	7.95±0.06
Microenca-psulated phage	0	4.31±0.42	6.62±0.35	7.31±0.43	7.75±0.38	7.76±0.02	7.77±0.01	7.77±0.01	7.77±0.01

Table S5. The activity of free phages stored at 4°C and 25°C

Temperature	0 week	1 week	2 week	3 week	4 week	5 week	6 week

**4℃**	8.00±0.30	7.88±0.25	7.46±0.35	7.04±0.30	6.48±0.45	5.83±0.23	5.12±0.34
**25℃**	8.00±0.30	7.39±0.27	6.58±0.34	5.68±0.35	4.86±0.26	3.82±0.31	2.38±0.45

Table S6. The activity of microencapsulated phages stored at 4℃ and 25℃

Temperature	0 week	1 week	2 week	3 week	4 week	5 week	6 week
**4℃**	8.00±0.30	7.96±0.08	7.94±0.07	7.92±0.08	7.91±0.09	7.89±0.07	7.87±0.09
**25℃**	8.00±0.30	7.87±0.06	7.75±0.08	7.62±0.09	7.42±0.08	7.21±0.07	6.98±0.10

Table S7. The number of *Salmonella* colonization in each group after the treatment of infected chicks with microencapsulated phages

Group	Duodenum	Jejunum	Ileum	Colorectal
**Control group**	2.10±0.15	2.38±0.16	2.42±0.18	4.11±0.23
**Infection group**	3.39±0.17	3.51±0.15	3.98±0.19	5.86±0.14
**Free phage group**	3.27±0.24	3.38±0.18	3.73±0.19	5.34±0.21
**Microencapsulated phage group**	2.84±0.22	2.88±0.17	3.32±0.19	4.62±0.18
